# *Coffea arabica* Extract Attenuates Atopic Dermatitis-like Skin Lesions by Regulating NLRP3 Inflammasome Expression and Skin Barrier Functions

**DOI:** 10.3390/ijms241512367

**Published:** 2023-08-02

**Authors:** Qiao-Xin Chang, Jia-Ling Lyu, Po-Yuan Wu, Kuo-Ching Wen, Chang-Cheng Chang, Hsiu-Mei Chiang

**Affiliations:** 1 Department of Cosmeceutics, China Medical University, Taichung 406, Taiwan; nancystar597@gmail.com (Q.-X.C.); u105306601@cmu.edu.tw (J.-L.L.); kcwen0412@gmail.com (K.-C.W.); changcc1975@gmail.com (C.-C.C.); 2 Ph.D. Program for Biotechnology Industry, China Medical University, Taichung 406, Taiwan; 3 Department of Dermatology, China Medical University Hospital, Taichung 404, Taiwan; wu.poyuan@gmail.com; 4 School of Medicine, China Medical University, Taichung 404, Taiwan

**Keywords:** *Coffea arabica*, atopic dermatitis, immune, inflammasome, skin barrier function, anti-oxidation, anti-inflammation

## Abstract

Atopic dermatitis (AD) is a common skin disease worldwide. The major causes of AD are skin barrier defects, immune dysfunction, and oxidative stress. In this study, we investigated the anti-oxidation and anti-inflammation effects of *Coffea arabica* extract (CAE) and its regulation of the skin barrier and immune functions in AD. In vitro experiments revealed that CAE decreased the reactive oxygen species levels and inhibited the translocation of nuclear factor-κB (NF-κB), further reducing the secretion of interleukin (IL)-1β and IL-6 induced by interferon-γ (IFN-γ)/tumor necrosis factor-α (TNF-α). Moreover, CAE decreased IFN-γ/TNF-α-induced NLR family pyrin domain-containing 3 (NLRP3), caspase-1, high-mobility group box 1 (HMGB1), and receptor for advanced glycation end products (RAGE) expression levels. It also restored the protein levels of skin barrier function-related markers including filaggrin and claudin-1. In vivo experiments revealed that CAE not only reduced the redness of the backs of mice caused by 2,4-dinitrochlorobenzene (DNCB) but also reduced the levels of pro-inflammatory factors in their skin. CAE also reduced transepidermal water loss (TEWL) and immune cell infiltration in DNCB-treated mice. Overall, CAE exerted anti-oxidation and anti-inflammation effects and ameliorated skin barrier dysfunction, suggesting its potential as an active ingredient for AD treatment.

## 1. Introduction

Atopic dermatitis (AD) is a skin condition with chronic relapsing inflammation, and its prevalence is high all over the world. The prevalence of AD is 0.96% to 22.6% in children and 1.2% to 17.1% in adults around the world [1]. The prevalence of AD varies in each country because of the various criteria of the disease. The progression of this skin disorder is affected by dysfunction of the epidermal barrier and immune system. AD is characterized by itchy eczematous lesions on the skin, especially the flexural areas [2]. It is not a serious disease but seriously affects life quality, including sleep disorders and anxiety. The etiology of AD is complex and related to many endogenous and exogenous factors [3], including environmental factors, genetic susceptibility, immunological imbalances, and skin barrier defects [4]. Many studies have focused on the dysregulation of T helper (Th) cells, production of immunoglobulin E (IgE), and hyperactivity of mast cells in the development of pruritus, inflammation, and AD [5]. However, skin barrier dysfunction plays a key role in AD development [6].

Skin is the primary defense organ in the human body against external stimuli, such as irritants, allergens, environmental pathogens, and radiation [7]. Keratinocytes, the major cell type in the epidermis, release pro-inflammatory cytokines and chemokines on exposure to immune triggers and participate in the progression of AD [8]. Immune system abnormalities in AD mainly involve Th cells, which recognize antigens and modulate immune responses. Naive T cells differentiate into type 1 and type 2 helper T cells (Th1 and Th2 cells, respectively) in the presence of different cytokines [9]. Co-stimulatory factors and cytokines affect the Th1/Th2 balance to promote the conversion of acute lesions to chronic lesions [10,11].

Skin barrier dysfunction drives disease activity in AD. AD is characterized by dry skin, including skin damage and increased TEWL. Filaggrin plays key structural and functional roles in the epidermis of the skin [12]. Filaggrin deficiency decreases the ability of keratinocytes to maintain hydration, causing pruritus and even AD in severe cases. Impaired barrier function facilitates the entry of allergens, resulting in an inflammatory response that eventually causes AD [13,14]. The main component of tight junctions in the epidermis is claudin-1, which is essential for barrier function and involved in the development of various human skin diseases, especially AD [15].

Topical corticosteroids are important anti-inflammatory drugs that alleviate redness, itching, and inflammation and act as the mainstream treatment for AD. However, corticosteroids may cause side effects, such as cutaneous atrophy, immunosuppression, and thinning of the skin, limiting their use [16]. Therefore, natural plant extracts have been explored for AD treatment. *Coffea arabica* (Rubiaceae) leaves, containing high levels of phenolic compounds, exhibit strong antioxidant activity and inhibit the severe acute respiratory syndrome coronavirus type 2 (SARS-CoV-2) infection [17,18]. We previously reported that *C. arabica* extract (CAE) containing chlorogenic acid (48.3 mg/g) and a small amount of caffeic acid exhibits antioxidant, anti-photoaging, and anti-inflammatory activities in human skin fibroblasts and null mice [19,20]. CAE inhibits ultraviolet (UV)-induced reactive oxygen species (ROS) generation, nuclear factor-κB (NF-κB) and interleukin-6 (IL-6) expression, and UV exposure-induced transepidermal water loss (TEWL) increment [20]. However, the specific roles and action mechanism of CAE in AD remain unclear. Therefore, in this study, we aimed to explore the efficacy and regulatory mechanisms of CAE in AD treatment using in vivo and in vitro experiments.

## 2. Results

### 2.1. CAE Mediates Cytotoxicity in HaCaT Cells

**Figure 1:**
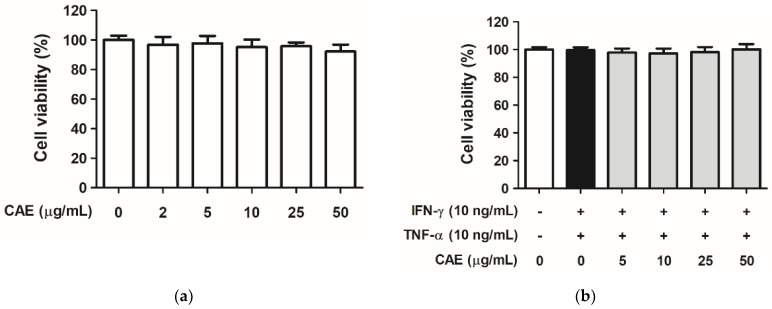
Cell viabilities of human skin keratinocytes after (**a**) *C. arabica* extract (CAE) treatment and (**b**) CAE treatment after IFN-γ and TNF-α induction for 24 h. CAE did not exhibit cytotoxic effects on HaCaT cells.

Human epidermal keratinocytes (HaCaT cells) were treated with CAE, and cell viability was measured using the 3-(4,5-dimethyl-2-thiazolyl)-2,5-diphenyl-2H-tetrazolium bromide (MTT) assay. As shown in Figure 1a, cell viability was more than 90% after treatment with CAE (2, 5, 10, 25, and 50 µg/mL), indicating that CAE did not exhibit cytotoxic effect on HaCaT cells. Then, the cells were pretreated with CAE (5, 10, 25, and 50 µg/mL) for 1 h and mixed with 10 ng/mL interferon (IFN)-γ and 10 ng/mL tumor necrosis factor (TNF)-α for 24 h. Neither the inducers (IFN-γ and TNF-α) nor CAE were found to be cytotoxic to HaCaT cells (Figure 1b).

### 2.2. CAE Attenuates Oxidative Stress in HaCaT Cells

#### 2.2.1. Effects of CAE on IFN-γ- and TNF-α-Induced Intracellular ROS Generation

**Figure 2:**
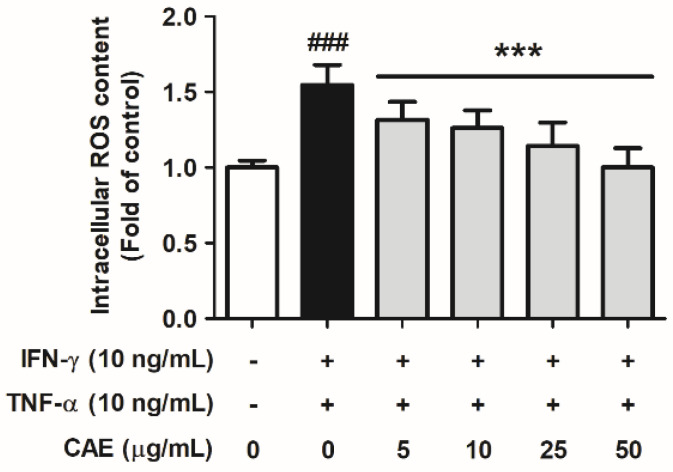
Effects of CAE on intracellular ROS levels in IFN-γ- and TNF-α-induced human skin keratinocytes. ###, *p* < 0.001 vs. non-induced group. ***, *p* < 0.001 vs. IFN-γ/TNF-α-induced group. CAE significantly attenuated IFN-γ- and TNF-α-induced oxidative stress in HaCaT cells.

HaCaT cells were pretreated with 5, 10, 25, and 50 µg/mL CAE for 1 h and treated with TNF-α and IFN-γ. As shown in Figure 2, IFN-γ and TNF-α increased the intracellular ROS levels by 1.54-fold compared with that in the control group. CAE (5, 10, 25, and 50 µg/mL) treatment decreased the intracellular ROS levels to 1.32-, 1.26-, 1.14- and 1.00-fold, respectively. These results suggest that CAE significantly attenuated IFN-γ- and TNF-α-induced oxidative stress.

#### 2.2.2. Effects of CAE on IFN-γ- and TNF-α-Induced Phospho (p)-Extracellular Signal-Regulated Kinase (ERK) and *p*-p38 Expression Levels

**Figure 3:**
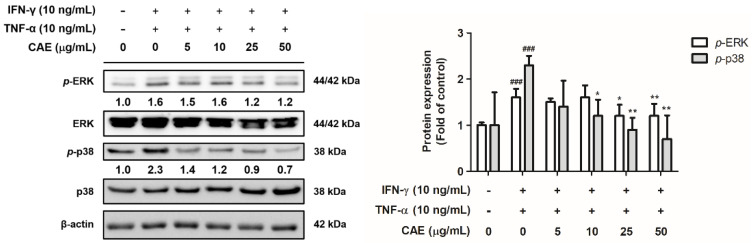
Effects of CAE on *p*-ERK and *p*-p38 protein expression levels in IFN-γ- and TNF-α-induced human skin keratinocytes after 24 h. ###, *p* < 0.001 vs. non-induced group. *, *p* < 0.05 and **, *p* < 0.01 vs. IFN-γ/TNF-α-induced group. CAE significantly reduced the IFN-γ- and TNF-α-induced *p*-ERK and *p*-p38 expression in HaCaT cells.

IFN-γ and TNF-α treatment significantly increased the expression levels of *p*-ERK and *p*-p38. CAE significantly reduced *p*-ERK expression at 25 µg/mL and *p*-p38 expression at 10 µg/mL (Figure 3).

### 2.3. CAE Reduces Inflammatory Signal Transduction in HaCaT Cells

#### 2.3.1. Effects of CAE on IFN-γ- and TNF-α-Induced NF-κB Nuclear Translocation

**Figure 4:**
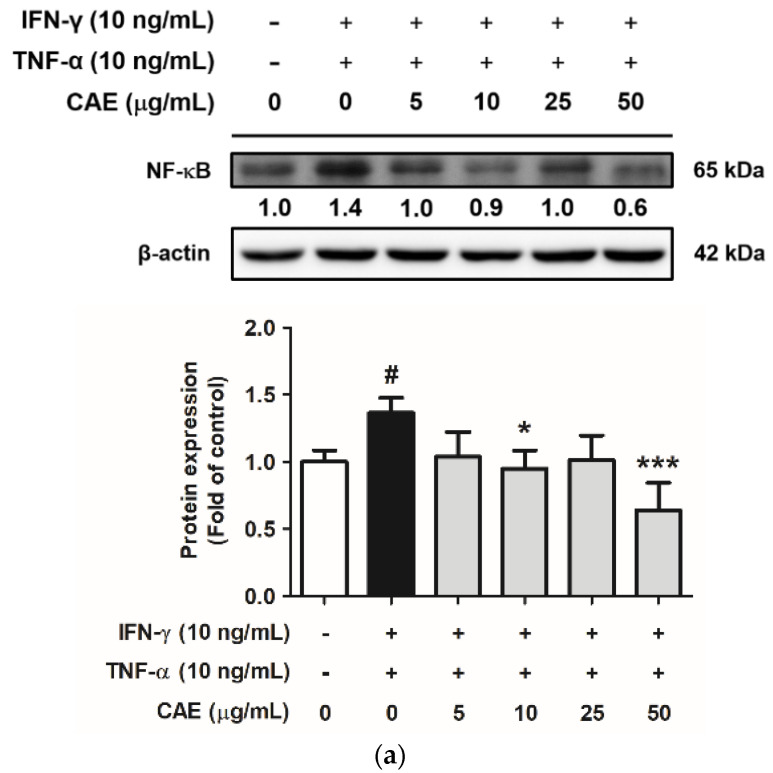

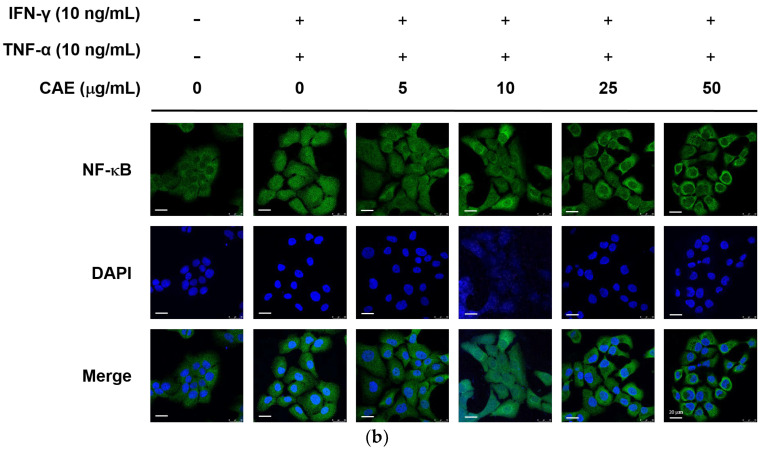
Effects of CAE on (**a**) protein expression levels and (**b**) nuclear translocation of NF-κB in IFN-γ- and TNF-α-induced human skin keratinocytes after 24 h. Immunofluorescence staining of NF-κB (green) and nucleus (DAPI, blue) (scale bar = 20 µm). #, *p* < 0.05 vs. non-induced group. *, *p* < 0.05 and ***, *p* < 0.001 vs. IFN-γ/TNF-α-induced group. CAE decreased the expression and the translocation of NF-κB stimulated by IFN-γ and TNF-α in HaCaT cells.

NF-κB, a transcription factor, mediates the production and secretion of pro-inflammatory factors involved in the development of several inflammatory diseases. In this study, the protein expression levels of NF-κB in the IFN-γ- and TNF-α-induced group increased 1.4-fold compared with that in the control group. Conversely, 50 µg/mL CAE decreased the protein expression of NF-κB by 0.6-fold compared to the control group (Figure 4a). Additionally, immunofluorescence staining demonstrated that IFN-γ and TNF-α stimulated the translocation of NF-κB into the cell nucleus, and this effect is inhibited by CAE (Figure 4b).

#### 2.3.2. Effects of CAE on IFN-γ- and TNF-α-Induced NLR Family Pyrin Domain-Containing 3 (NLRP3) Signaling Pathway

**Figure 5:**
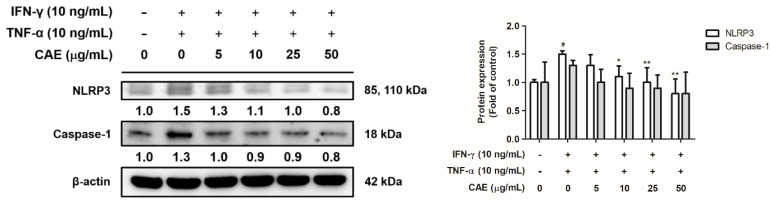
Effects of CAE on the NLR family pyrin domain-containing 3 (NLRP3) and caspase-1 protein expression levels in IFN-γ- and TNF-α-induced human skin keratinocytes after 24 h. #, *p* < 0.05 vs. non-induced group. *, *p* < 0.05 and **, *p* < 0.01 vs. IFN-γ/TNF-α-induced group. CAE decreased the expression of NLRP3 and caspase-1 stimulated by IFN-γ and TNF-α in HaCaT cells.

NLRP3 inflammasome is activated by oxidative stress and promotes the secretion of pro-inflammatory cytokines. In this study, the protein expression levels of NLRP3 in the IFN-γ- and TNF-α-induced group increased by 1.5-fold compared with that in the control group. Treatment with 10 µg/mL CAE decreased the protein expression of NLRP3 by 1.1-fold compared with the protein expression in the control group (Figure 5). In addition, IFN-γ and TNF-α treatment increased caspase-1 levels by 1.3-fold compared with the protein expression in the control group, and this activity was reversed after CAE treatment with no statistical difference (Figure 5).

#### 2.3.3. Effects of CAE on IFN-γ- and TNF-α-Induced IL-1β and IL-6 Cytokines Secretion

**Figure 6:**
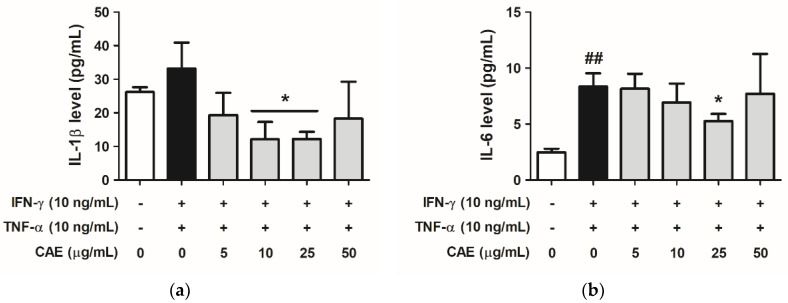
Effects of CAE on the secretion of (**a**) interleukin (IL)-1β and (**b**) IL-6 in IFN-γ- and TNF-α-induced human skin keratinocytes after 24 h. ##, *p* < 0.01 vs. non-induced group. *, *p* < 0.05 vs. IFN-γ/TNF-α-induced group. CAE decreased the IFN-γ- and TNF-α-induced IL-1β and IL-6 levels in HaCaT cells.

As shown in Figure 6a, IL-1β levels secreted from keratinocytes increased to 33.13 pg/mL (1.27-fold compared with that in the control group) in the IFN-γ- and TNF-α-induced group and decreased to 19.29, 12.12, 12.22, and 18.07 pg/mL at 5–50 µg/mL in the CAE-treated group. Similarly, IL-6 levels were elevated to 8.34 pg/mL (3.38-fold compared with that in the control group) after treatment with IFN-γ and TNF-α; however, treatment with CAE (5–50 µg/mL) decreased the levels of IL-6 (Figure 6b).

#### 2.3.4. Effects of CAE on IFN-γ- and TNF-α-Induced High-Mobility Group Box 1 (HMGB1) and Receptor for Advanced Glycation End Products (RAGE) Expression Levels

**Figure 7:**
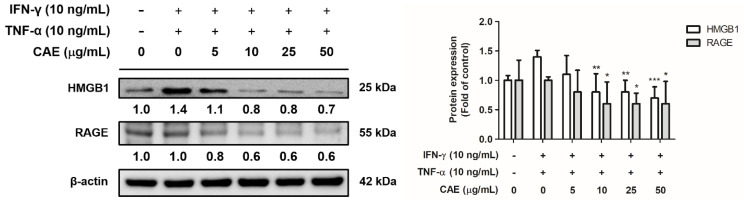
Effects of CAE on HMGB1 and RAGE protein expression levels in IFN-γ- and TNF-α-induced human skin keratinocytes after 24 h. *, *p* < 0.05, **, *p* < 0.01 and ***, *p* < 0.001 vs. IFN-γ/TNF-α-induced group. CAE significantly reduced the IFN-γ- and TNF-α-induced HMGB1 and RAGE expression in HaCaT cells.

Treatment with IFN-γ and TNF-γ significantly increased the expression levels of HMGB1 and RAGE. CAE significantly reduced HMGB1 expression at 10 µg/mL and RAGE expression at 10 µg/mL (Figure 7).

### 2.4. CAE Restores the Skin Barrier Function-Related Marker Expression in HaCaT Cells

**Figure 8:**
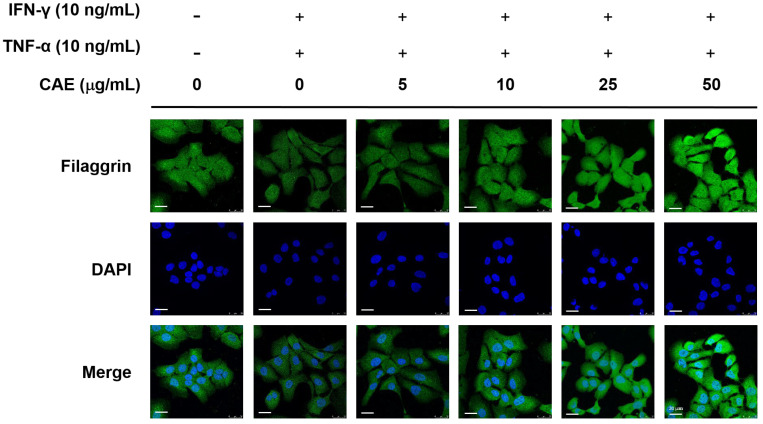
Effects of CAE on the nuclear translocation of filaggrin in IFN-γ- and TNF-α-induced human skin keratinocytes after 24 h. Immunofluorescence staining of filaggrin (green) and nucleus (DAPI, blue) (scale bar = 20 µm). CAE increased the translocation of filaggrin stimulated by IFN-γ and TNF-α in HaCaT cells.

AD is characterized by dry skin and impaired skin barrier function associated with increased TEWL. Filaggrin and claudin-1 are key factors in the epidermal cell barrier that regulate the Th1/Th2 inflammatory response. Immunofluorescence staining demonstrated that IFN-γ and TNF-α decreased the fluorescence of filaggrin, whereas CAE reversed this effect (Figure 8).

**Figure 9:**
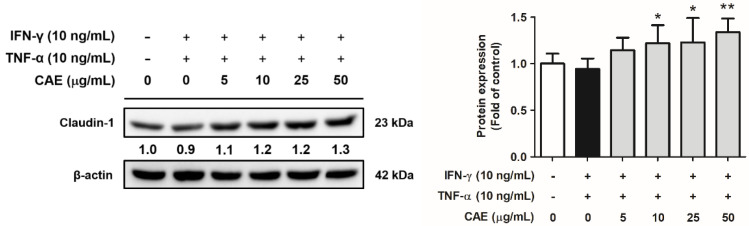
Effects of CAE on claudin-1 protein expression levels in IFN-γ- and TNF-α-induced human skin keratinocytes after 24 h. *, *p* < 0.05 and **, *p* < 0.01 vs. IFN-γ/TNF-α-induced group. CAE increased the expression of claudin-1 stimulated by IFN-γ and TNF-α in HaCaT cells.

As shown in Figure 9, IFN-γ and TNF-α decreased the protein levels of claudin-1 by 0.9-fold in HaCaT cells compared with that the protein expression in the control group, whereas CAE (5–50 µg/mL) increased the expression levels of claudin-1 by 1.1-, 1.2-, 1.2-, and 1.3-fold, respectively. These results suggest that CAE restores the skin barrier function-related protein expression.

### 2.5. CAE Improves 2,4-Dinitrochlorobenzene (DNCB)-Induced AD-like Skin Lesions in Mice

#### 2.5.1. Effects of CAE on DNCB-Induced Body Weights

**Figure 10:**
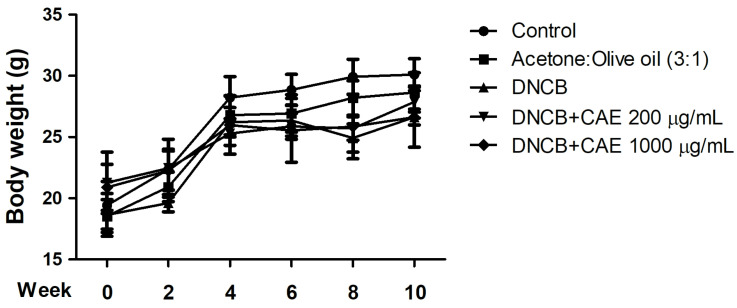
Body weights of BALB/c mice during 10 weeks of treatment. Non-significant difference between the groups.

Body weight changes in the mice of each group over 10 weeks are shown in Figure 10. The body weight of each group increased steadily over time, with no significant difference between the groups.

#### 2.5.2. Effects of CAE on DNCB-Induced a* Values

**Figure 11:**
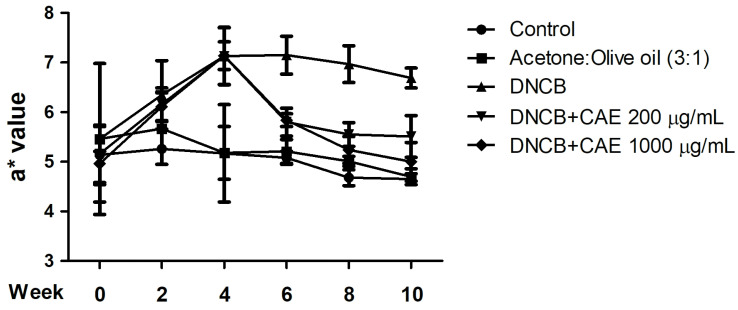
Effects of CAE inflammation indicated by a* values in DNCB-induced BALB/c mice during 10 weeks of treatment. CAE improved DNCB-induced erythema and inflammation in AD.

The a* value is an indication of the redness and swelling of the skin and also an index of skin inflammation. As shown in Figure 11, the a* value for each group was approximately 4–5 in the 0th week. The a* value of the DNCB-induced group was increased in the 4th week and maintained at 6–7 until the 10th week. However, 200 and 1000 µg/mL CAE reduced the a* value from the 6th week of treatment, similar to the results in the control group. Based on the results, CAE improved DNCB-induced erythema and inflammation in AD.

#### 2.5.3. Effects of CAE on DNCB-Induced Ear Thickness

**Figure 12:**
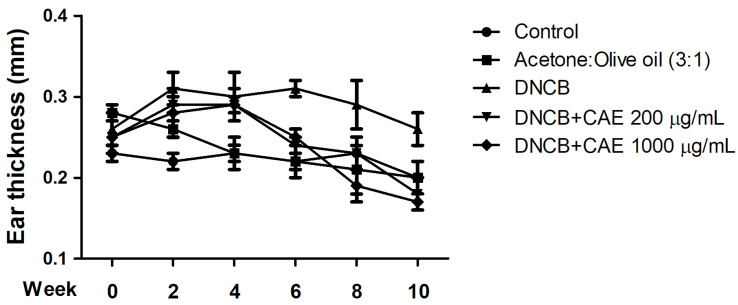
Effects of CAE on ear thickness in DNCB-induced BALB/c mice during 10 weeks of treatment. CAE inhibited DNCB-induced skin edema.

Ear thickness of each mice group was measured at the 0th, 2nd, 4th, 8th, and 10th weeks, and the results are shown in Figure 12. On the 0th week, the ear thickness in each group was approximately 0.2–0.3 mm. From the 6th week, a difference was observed in the ear thickness of DNCB-induced and CAE (200 and 1000 µg/mL)-treated mice. These results suggest that CAE inhibited DNCB-induced skin edema.

#### 2.5.4. Effects of CAE on DNCB-Induced TEWL

**Figure 13:**
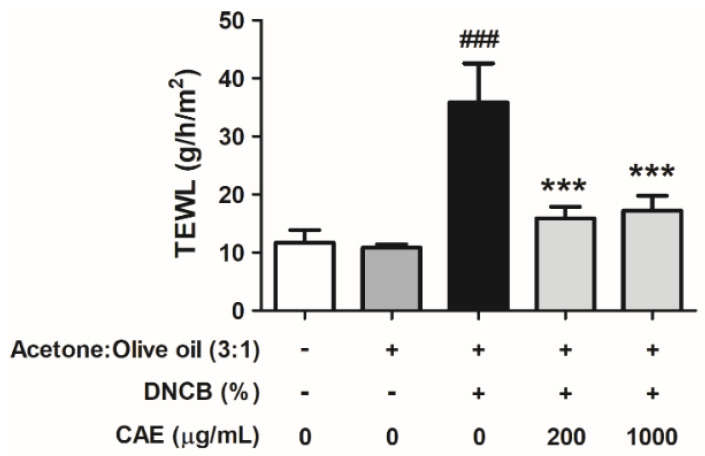
Effects of CAE on TEWL in DNCB-induced BALB/c mice in the 10th week of treatment. ###, *p* < 0.001 vs. control group. ***, *p* < 0.001 vs. DNCB-induced group. CAE ameliorated DNCB-induced skin barrier dysfunction.

Skin barrier function damage leads to elevated TEWL. As shown in Figure 13, TEWL was 11.7 ± 2.2 g/h/m^2^ in the control group mice and significantly increased to 35.9 ± 6.7 g/h/m^2^ in the DNCB-induced group. CAE (200 and 1000 µg/mL) treatment yielded TEWL values of 15.9 ± 2.0 and 17.2 ± 2.5 g/h/m^2^, respectively. These results suggest that CAE ameliorated DNCB-induced skin barrier dysfunction.

#### 2.5.5. Effects of CAE on DNCB-Induced Chemokine and Cytokine Levels

**Figure 14:**
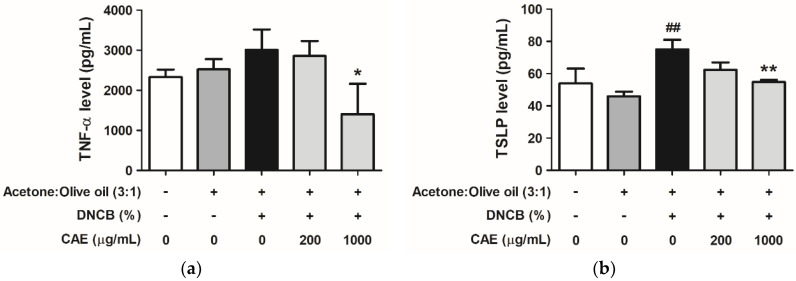
Effects of CAE on the secretion of (**a**) TNF-α and (**b**) TSLP in DNCB-induced BALB/c mice in the 10th week of treatment. ##, *p* < 0.01 vs. control group. *, *p* < 0.05 and **, *p* < 0.01 vs. DNCB-induced group. CAE decreased DNCB-induced TNF-α and TSLP levels.

TNF-α and thymic stromal lymphopoietin (TSLP) are potential biomarkers for Th cell-driven inflammation in AD. Here, we determined their levels using ELISA kits. As shown in Figure 14a, TNF-α levels were increased in the DNCB-treated group (1.29-fold compared with that in the control group) and decreased in the CAE (200 and 1000 µg/mL)-treated groups (1.22- and 0.60-fold compared with that in the control group, respectively). TSLP levels in the DNCB-treated group increased to 74.96 pg/mL (1.39-fold compared with that in the control group) and decreased to 62.28 and 54.76 pg/mL in the 200 and 1000 µg/mL CAE-treated groups, respectively (Figure 14b).

#### 2.5.6. Effects of CAE on DNCB-Induced Changes in Skin Pathology

**Figure 15:**
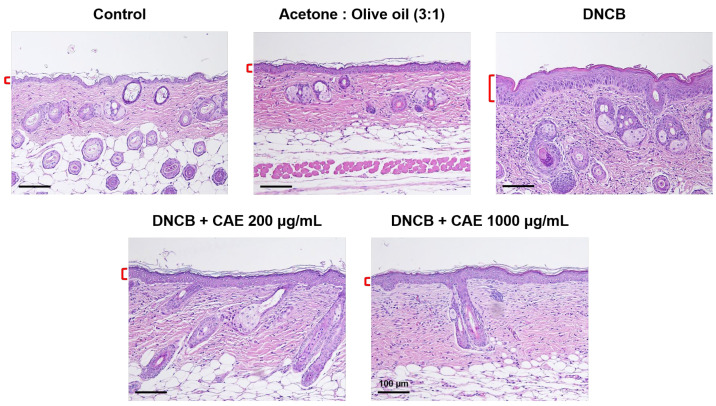
Histopathological characterization of H&E-stained BALB/c mice treated with DNCB and CAE (scale bar = 100 µm). The red mark represents the thickness of the skin epidermis CAE decreased DNCB-induced skin epidermal thickness.

**Figure 16:**
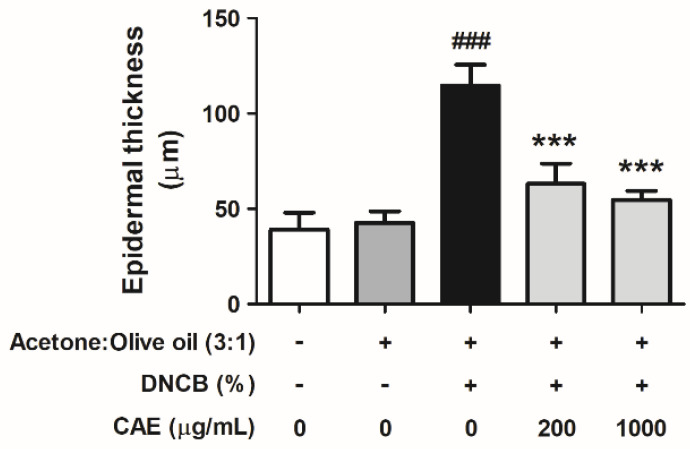
Effects of CAE on the epidermal thickness of H&E-stained DNCB-induced BALB/c mice in the 10th week of treatment. ###, *p* < 0.001 vs. control group. ***, *p* < 0.001 vs. DNCB-induced group. CAE significantly decreased DNCB-induced epidermal thickness.

Hematoxylin and eosin (H&E) staining revealed the infiltration of eosinophils and neutrophils into the skin dermis (Figure 15). In addition to the infiltration of some immune cells, H&E-stained images showed changes in the skin epidermal thickness. Epidermal thickness increased in the DNCB-treated group and significantly decreased after treatment with CAE (Figure 16).

**Figure 17:**
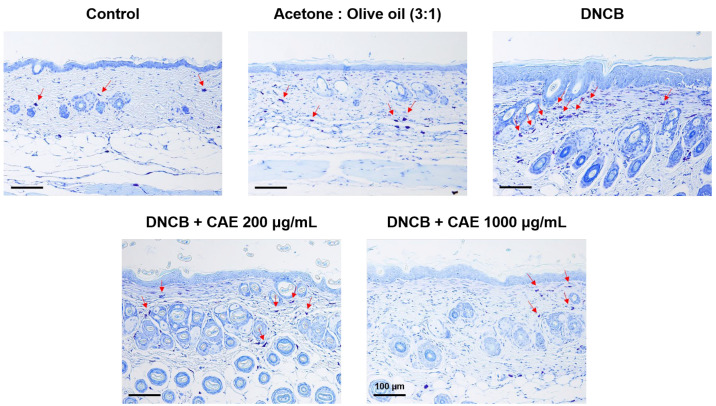
Histopathological characterization (indicated by arrows) of toluidine blue-stained BALB/c mice treated with DNCB and CAE (scale bar = 100 µm). CAE attenuated DNCB-induced AD-like skin lesions.

Mast cells are involved in the exacerbation of inflammatory diseases. As shown in Figure 17, toluidine blue staining revealed that the number of mast cells increased in the DNCB-treated group and decreased after CAE treatment. These results suggest that CAE attenuated DNCB-induced AD-like skin lesions.

## 3. Discussion

AD is a common, recurrent, and chronic inflammatory disease characterized by skin barrier dysfunction, inflammation, and chronic itching [21,22]. AD is also an immune system skin disorder, and the secretion of cytokines, especially IL-4, IL-12, and IL-13, causes barrier defects and inflammation, resulting in the clinical features of eczema [23,24]. Several factors associated with immunity, inflammation, and oxidative stress may cause AD. Several theories have been proposed regarding the mechanisms underlying AD. Currently, studies are investigating the roles of the immune system, skin structural gene mutations, defects in skin cells (keratinocytes), skin surface microbiome (bacteria, viruses, and yeasts), and other factors in AD [23]. Regulation of skin barrier function plays an important role in skin disorders. Corticosteroids and some immunosuppressants are used to treat AD, but the long-term use of these drugs causes adverse effects. Therefore, natural products with anti-oxidation and anti-inflammation activities may be good alternatives for the treatment of AD.

Exposure of the skin to environmental pollutants, allergens, and pathogens generates ROS, which induces intracellular oxidative stress and promotes the development and progression of skin disorders, including intrinsic and extrinsic aging, cancer, and dermatitis [25,26]. Antioxidant effects of natural substances can effectively inhibit skin inflammation and oxidative stress-induced disorders [27,28]. Ursolic acid inhibits ROS accumulation and the inflammatory response in TNF-α/IFN-γ-stimulated HaCaT cells to improve the symptoms of AD [29]. We previously showed that CAE exhibits a strong antioxidant effect by scavenging free radicals and reducing UV-induced ROS generation in human skin fibroblasts [17,19]. In addition, CAE elevates the levels of oxidative stress defense enzymes, including catalase, to ameliorate UVB-induced inflammation and skin damage [20]. In this study, CAE decreased IFN-γ/TNF-α-induced intracellular ROS levels to attenuate oxidative stress in human skin keratinocytes, resulting in the alleviation of AD.

Inflammasomes are protein complexes that cleave pro-inflammatory cytokines into active forms [30]. Changes in the microenvironment regulate the initiation of the NLRP3 inflammasome, which modulates caspase-1, contributing to IL-1β and IL-18 maturation [31]. IL-1β has been suggested to contribute to numerous skin inflammatory diseases, such as AD and psoriasis [32]. As shown in Figure 5 and Figure 6, CAE decreased not only the protein expression of NLRP3 but also the translocation of NF-κB into the cell nucleus and reduced the secretion of IL-1β and IL-6 induced by IFN-γ and TNF-α. In addition, in vivo experiments revealed that CAE lowered the a* values (redness) and TNF-α and TSLP contents in mouse skin induced by DNCB, indicating that CAE alleviated DNCB-induced AD-like skin lesions via anti-inflammation activity. Our previous study reported that CAE inhibits UV-induced cyclooxygenase-2 (COX-2) and inducible nitric oxide synthase (iNOS) expression in human fibroblasts and UV-induced NF-κB and IL-6 overexpression and TEWL in hairless mice [20]. CAE also reduced the translocation of NF-κB into the nucleus of fibroblasts induced by UVB [20]. Therefore, CAE may ameliorate the inflammatory responses and symptoms of AD.

HMGB1 is an intranuclear architectural protein that acts as a potent pro-inflammatory mediator. In inflammatory skin diseases, such as AD and psoriasis, HMGB1 acts as an endogenous danger signal that reacts with receptors, such as RAGE and Toll-like receptors (TLRs), to stimulate cytokine production [33]. Activated RAGE transmits cell surface signals to induce the secretion of ILs via modulation of the mitogen-activated protein kinase and NF-κB pathways through RAGE, promoting inflammation [34,35]. TLR4/NF-κB pathway is closely related to the development of AD, and NF-κB is a major transcription factor involved in the inflammatory response of AD [36]. Here, CAE treatment reduced IFN-γ/TNF-α-induced expression of HMGB1, RAGE, NF-κB, *p*-ERK, and *p*-p38 proteins to decrease inflammation in mice.

As a barrier to the body and the outside world, the skin maintains the water content and temperature in the body to prevent water loss and dermal penetration of allergens and microbiota. AD is characterized by dry skin and is associated with increased TEWL [6]. Therefore, the degree of epidermal damage can be estimated using the TEWL value [37,38]. Filaggrin and claudin-1 are important factors in skin barrier function [39]. Filaggrin controls TEWL and preserves stratum corneum hydration to regulate cell differentiation, pro-inflammatory cytokine expression, and skin barrier function [40]. In our study, Figure 8 and Figure 9 showed that CAE increased claudin-1 and filaggrin expression that was reduced by IFN-γ/TNF-α. The results of in vivo experiments showed that the TEWL value of the mouse back skin was elevated in the DNCB-induced group and decreased after treatment with CAE. This indicated that CAE effectively restored skin barrier dysfunction. In our previous study, long-term UVB exposure increased TEWL in mice; however, CAE reversed this effect [20]. Therefore, CAE restored the expression of barrier function-related proteins and protected skin from damage induced by external factors. Tight junctions regulate the permeability of water, ions, and water-soluble molecules to maintain skin hydration. Overexpression of IFN-γ in the lesional skin leads to a decrease in claudin-1 expression in mice. Claudin-1-deficient mice die on the first day after birth and show significant wrinkles, severe dehydration, and increased TEWL [41]. De Benedetto et al., showed that the expression of claudin-1 was inversely related to the total eosinophil count and total IgE levels in serum [42]. Therefore, defects in tight junctions may be associated with enhanced Th2 inflammatory response. In this study, our results suggest that CAE increases filaggrin and claudin-1 protein expression to maintain the water content of the dorsal skin and the integrity and function of the stratum corneum in mice with AD-like lesions.

## 4. Materials and Methods

### 4.1. Chemicals and Reagents

Fetal bovine serum and Dulbecco’s Modified Eagle’s Medium (DMEM), penicillin, streptomycin used in the cell culture, and ProLong Diamond Antifade Mountant with 4′,6-diamidino-2-phenylindole (DAPI) were purchased from Gibco, Thermo Fisher Scientific, Inc. (Billings, MT, USA). IFN-γ was purchased from BioVision, Inc. (Milpitas, CA, USA) and TNF-α antibody was purchased from Signalway Antibody LLC (Greenbelt, MD, USA). Paraformaldehyde, 2′,7′-dichlorofluorescin diacetate (DCFH-DA), and DNCB were obtained from Sigma-Aldrich Chemical Corporation (St. Louis, MO, USA). MTT, sodium dodecyl sulfate (SDS), and Triton X-100 were purchased from USB Corporation (Douglasville, AG, USA). Acetone and isopropanol were purchased from J.T. Baker Co. (Phillipsburg, NY, USA). Western Bright ECL kit was purchased from Advansta Inc. (Menlo Park, CA, USA). The chemicals used in the experiments were of reagent grade. Antibodies including human IL-1β (EH0185), human IL-6 (EH0201), mouse TNF-α (EM0183), and mouse TSLP (EM0201) ELISA kits were purchased from Wuhan Fine Biotech Co., Ltd. (Wuhan, China).

### 4.2. Preparation and Quantitative Analysis of CAE

The fresh coffee leaves (10 g) were collected in Yunlin County in Taiwan and extracted with 600 mL methanol. The extraction yield of CAE was 10.4%. The extract was quantitatively analyzed using the high-performance liquid chromatography-UV/Vis method and the amount of chlorogenic acid was 48.3 ± 0.4 mg/g, as previously reported [19].

### 4.3. In Vitro Model

#### 4.3.1. Cell Viability Test

HaCaT cells were seeded in a 24-well plate at a density of 2 × 10^5^ cells/well and incubated for 24 h. The culture medium was aspirated, mixed with different concentrations of CAE, and incubated for 24 h, after which MTT solution was added to each well. The concentration of CAE used in this study referred to the concentration used in our previous study [19]. After 3 h, the MTT solution was aspirated, and an isopropanol solution was added. Absorbance was measured at 570 nm using a microplate reader (Sunrise, Tecan, Austria), as previously described [27,43].

#### 4.3.2. Intracellular ROS Scavenging Assay

HaCaT cells were seeded in a 24-well plate (2 × 10^5^ cells/well) and incubated for 24 h. The culture medium was aspirated, mixed with different concentrations of CAE, and incubated for 1 h. Then, the HaCaT cells were mixed with the inducing substances, 10 ng/mL IFN-α, and 10 ng/mL TNF-α for 24 h, washed with phosphate-buffered saline (PBS), and mixed with 500 µL of DCFDA reagent for 30 min. The fluorescence intensity (488 nm excitation and 520 nm emission) was measured using a microplate reader (Thermo Electron Corporation, Vantaa, Finland), as previously described [27,43].

#### 4.3.3. Western Blotting

HaCaT cells were seeded into a 10-cm dish at a density of 1.5 × 10^6^ cells/dish and cultured for 24 h. Various concentrations of CAE were treated and maintained in the incubator for 1 h, after which IFN-γ (10 ng/mL) and TNF-α (10 ng/mL) were added and incubated for 24 h. Then, the culture solution was removed and cells were washed with PBS, collected, and homogenized in a lysis buffer. The cell lysates were incubated on ice for 30 min, centrifuged at 12,000× *g* for 15 min, and the supernatant was transferred to a microtube.

The prepared samples containing equal amounts of protein were separated via SDS-polyacrylamide gel electrophoresis, transferred to a polyvinylidene fluoride membrane, blocked with 5% non-fat milk prepared in TBST buffer, and incubated for non-specific antibody binding at room temperature. The membrane was washed with TBST buffer and incubated with specific primary antibodies overnight at 4 °C overnight. Subsequently, the antibodies were removed, the PVDF membrane was washed with TBST three times, and a secondary antibody was added. Finally, the membrane was incubated with chemiluminescent reagents and visualized using LAS-4000 (Fujifilm, Tokyo, Japan). Quantitative analysis of the blots was performed using the ImageJ software (https://imagej.nih.gov/ij/, National Institutes of Health, Bethesda, MD, USA).

#### 4.3.4. Immunofluorescence Staining

HaCaT cells were seeded in a 6-well plate (3 × 10^5^ cells/well) and incubated for 24 h. Cells were pretreated with different concentrations of CAE for 1 h and mixed with IFN-γ (10 ng/mL) and TNF-α (10 ng/mL) for 24 h. Then, the culture medium was removed, cells were washed with PBS, and 4% paraformaldehyde was added for 30 min to fix the cells. Next, the cells were incubated with 5% non-fat milk with 0.3% Triton X-100 for 1 h. Then, cells were incubated with primary antibodies, covered with paraffin at 4 °C overnight, and washed with PBS, followed by incubation with the secondary antibody. The slides were mounted with ProLong Gold Antifade reagent and observed using a confocal microscope (Leica Microsystems, Wetzlar, Hesse, Germany), as previously described [27,43].

#### 4.3.5. Secreted Protein Measurement

IL-1β and IL-6 levels were measured. The culture medium was centrifuged at 12,000× *g* for 15 min and the supernatant was added to a capture antibody-pre-coated 96-well plate. After incubation for 90 min at 37 °C, the medium was removed and washed twice with the wash buffer. Biotin-labeled antibody working solution (100 µL) was added to the wells for 60 min and washed with the wash buffer. Horseradish peroxidase-streptavidin conjugate solution was added for 30 min, and the unbound conjugates were washed away with the wash buffer. TMB substrates were catalyzed with HRP to produce a blue product, which changed color to yellow after the addition of an acidic stop solution to visualize the HRP enzymatic reaction. The intensity of the yellow color was proportional to the target level of the sample captured on the plate, and the absorbance was read at 450 nm using a multimode reader (Synergy HTX, BioTek Instruments, Winooski, VT, USA).

### 4.4. In Vivo Model

#### 4.4.1. Experimental Design

Five-week-old BALB/c female mice were bred under relative humidity (50 ± 10%), controlled temperature (24 ± 2 °C), and a 12 h light/dark cycle. Mice maintained a standard rodent chow Prolab RMH 2500, 5P14 (12% kcal from fat, 60% kcal from carbohydrate, and 28% kcal from protein) (Lab Diet, St. Louis, MO, USA), and drank water ad libitum during the experiment. Mice (average initial weight: 19.8 ± 1.3) were randomly divided into five groups: control (without treatment), inducer vehicle (acetone:olive oil = 3:1), DNCB (DNCB prepared in 3:1 acetone/olive oil solution), low-dose (DNCB + 200 µg/mL CAE), and high-dose (DNCB + 1000 µg/mL CAE) groups. The doses of CAE used in this study referred to the doses used in our previous study [19]. An electric razor and hair removal cream was used weekly to remove the back hair of the mice, and DNCB solution was applied to the back hairless area and ears of the mice to induce immunological symptoms and AD-like skin lesions.

In the first week, 200 µL of 1% DNCB solution was applied to the back hairless area and 10 µL of 1% DNCB solution was applied to the ears thrice a week (2, 4, and 6 d after hair removal). From the second week, 200 μL of 0.2% DNCB solution was topically applied to the hairless area and 10 μL of 0.2% DNCB solution was applied to the ears thrice a week for nine weeks.

#### 4.4.2. Body Weight Measurement

The body weights of the mice were measured and recorded every two weeks on the first day before the start of the experiment.

#### 4.4.3. a* Value Measurement

The day after the last administration of DNCB every two weeks, the degree of redness (a* value) of mouse ears induced by DNCB was measured via spectrophotometry (SCM-104/108; Ruyico Technology Corporation, Taipei, Taiwan). The higher the a* value, the redder is the skin [44].

#### 4.4.4. Ear Thickness Measurement

The day after the last administration of DNCB every two weeks, the degree of swelling in the mouse ears induced by DNCB was measured using a digital caliper.

#### 4.4.5. TEWL Measurement

After 10 weeks of the experiment, Tewameter TM 300 of the Multi-Function Skin Tester MPA580 (Courage+Khazaka Electronic GmbH, Cologne, Germany) was applied to measure the TEWL of the mice on the last day [44].

#### 4.4.6. Determination of Chemokine and Cytokine Levels

After sacrifice, the back skin of the mice was cut, the skin tissue was homogenized in PBS containing protease inhibitors on ice, and the homogenates were centrifuged at 5000× *g* for 5 min to obtain the supernatant. Then TNF-α and TSLP levels were determined and the absorbance was measured at 450 nm by using a multi-mode reader (Synergy HTX, BioTek Instruments, Winooski, VT, USA).

#### 4.4.7. Evaluation of Skin Pathology Changes

After sacrifice, the back skin of the mice was fixed with formalin, embedded in paraffin, sectioned, and stained with H&E and toluidine blue. Images were captured using an optical microscope with a camera.

### 4.5. Statistical Analyses

The final results of the experiments are expressed as the mean ± standard deviation calculated from at least three independent tests. All experimental data analyses were conducted using GraphPad Prism 5 (GraphPad Software Inc., San Diego, CA, USA) for a one-way analysis of variance, followed by Tukey’s post hoc test. *p* < 0.05 was considered statistically significant.

## 5. Conclusions

In this study, CAE exhibited high antioxidant activity and eliminated the oxidative stress induced by IFN-γ/TNF-α. Furthermore, it inhibited inflammation and increased filaggrin and claudin-1 expression levels to protect the skin barrier. Animal experiments revealed that CAE exhibited strong anti-inflammatory effects, improved skin barrier functions, and decreased the infiltration of immune cells in mice. Our results suggest CAE as a potential active ingredient for AD treatment and it may apply to clinical trials for further understanding the activity of CAE on people suffering from AD.

## Data Availability

Not applicable.
